# Dynamic Chromatin Localization of Sirt6 Shapes Stress- and Aging-Related Transcriptional Networks

**DOI:** 10.1371/journal.pgen.1002153

**Published:** 2011-06-30

**Authors:** Tiara L. A. Kawahara, Nicole A. Rapicavoli, Angela R. Wu, Kun Qu, Stephen R. Quake, Howard Y. Chang

**Affiliations:** 1 Howard Hughes Medical Institute and Program in Epithelial Biology, Stanford University School of Medicine, Stanford, California, United States of America; 2 Howard Hughes Medical Institute and Department of Bioengineering, Stanford University School of Medicine, Stanford, California, United States of America; The University of North Carolina at Chapel Hill, United States of America

## Abstract

The sirtuin Sirt6 is a NAD-dependent histone deacetylase that is implicated in gene regulation and lifespan control. Sirt6 can interact with the stress-responsive transcription factor NF-κB and regulate some NF-κB target genes, but the full scope of Sirt6 target genes as well as dynamics of Sirt6 occupancy on chromatin are not known. Here we map Sirt6 occupancy on mouse promoters genome-wide and show that Sirt6 occupancy is highly dynamic in response to TNF-α. More than half of Sirt6 target genes are only revealed upon stress-signaling. The majority of genes bound by NF-κB subunit RelA recruit Sirt6, and dynamic Sirt6 relocalization is largely driven in a RelA-dependent manner. Integrative analysis with global gene expression patterns in wild-type, *Sirt6−/−,* and double *Sirt6−/− RelA−/−* cells reveals the epistatic relationships between Sirt6 and RelA in shaping diverse temporal patterns of gene expression. Genes under the direct joint control of Sirt6 and RelA include several with prominent roles in cell senescence and organismal aging. These data suggest dynamic chromatin relocalization of Sirt6 as a key output of NF-κB signaling in stress response and aging.

## Author Summary

Sirtuins (Sirt) are a family of enzymes that modify chromatin and other proteins to affect gene activity. Loss of Sirt6 leads to a progeria-like phenotype in mice, but the target of SIRT6 action has been elusive. Here we show that Sirt6 binds to thousands of gene promoters in a stress-inducible fashion, guided by the stress-responsive transcription factor NF-κB. Both the departure and arrival of Sirt6 alter gene expression, shaping the temporal dynamics of NF-κB transcriptional response and directly controlling the expression of other key regulators of aging. These findings provide the first view of how an oscillatory transcription factor can drive a progression of chromatin changes over time.

## Introduction

Silent Information Regulator-2 (*Sir2*) encodes an NAD-dependent histone deacetylase that links chromatin regulation to genomic stability, gene silencing and lifespan in yeast. Sir2 deacetylates lysines in the amino terminal tails or histones H3 and H4 and in the globular core of histone H3 [1]–[4]. Mutations disrupting Sir2 mutation leads to global hyperacetylation of histones H3 and H4. Increased rDNA recombination and dysfunctional mating type loci silencing occurs, thus contributing to accelerated cellular aging and reduced replicative lifespan in yeast. Conversely, enhanced Sir2 function in several model organisms can increase lifespan [5].

Seven Sir2 homologues exist in the mammalian genome, termed sirtuins (SIRT1-7) [6]–[7]. The chromatin-associated sirtuin, SIRT6, is an important regulator of gene expression and genome integrity [8]–[13]. SIRT6 specifically deacetylates lysine 9 and 56 of histone H3 (H3K9Ac and H3K56Ac), and its deacetylase activity is involved in inhibition of gene expression [9]–[10], [12]. The NF-κB subunit RELA can recruit SIRT6 via direct protein-protein interaction to the promoters of several NF-κB target genes. SIRT6 then deacetylates H3K9Ac and destabilizes RELA occupancy, leading to termination of NF-κB-dependent gene expression [10]. It is unclear whether this model applies generally to most or to only select NF-κB target genes. Recently, it has been demonstrated that SIRT6 can act as a corepresser of the transcription factor, Hif1a, suggesting that SIRT6 may interact with additional regulators to modulate gene expression in a variety of contexts [12]. Additionally, the full set of genes targeted by SIRT6 remains to be determined.

NF-κB comprises a family of transcription factors that control the expression of genes involved in cells survival, senescence, inflammation, immunity and aging [14]. NF-κB proteins are responsive to stress signals including infection, inflammation, DNA damage, oxidative stress and metabolic stress. In response to such signals, the IκB kinase (IKK) complex phosphorylates the IκB proteins which bind and sequester NF-κB proteins in the cytoplasm. Phosphorylated IκB is subsequently ubiquitinated and degraded, and liberated NF-κB translocates into the nucleus to activate transcription of its target genes, including regulators of the NF-κB pathway [15]–[16]. As a result of negative feedback loops, NF-κB shuttles in and out of the nucleus, and target gene expression can be oscillatory. Intriguingly, subsequent rounds of NF-κB can activate different target genes due to chromatin changes induced by the pioneering round of NF-κB activation [17]–[18].

Disruption of *Sirt6* in mice results in a degenerative phenotype resembling premature aging [11]. Importantly, concomitant heterozygous knockout of RelA allows a significant fraction of mice to overcome the degenerative phenotypes and avoid lethality [10]–[11]. This genetic epistasis supports a model where Sirt6 limits excessive NF-κB-dependent transcription in order to promote longevity. NF-κB activity also increases with age in mice and humans, and is required to enforce cellular senescence and tissue aging [19]. Genes jointly controlled by Sirt6 and NF-κB should include important contributors to aging, but to date, the identity of relevant target genes are not known. In this respect, we determine the targets of Sirt6 genome-wide, reveal dynamic movement of Sirt6 during stress signaling and identify joint target genes, many of which are linked to aging.

## Results

### Dynamic relocalization of Sirt6 genome-wide upon stress signaling

**Figure 1:**
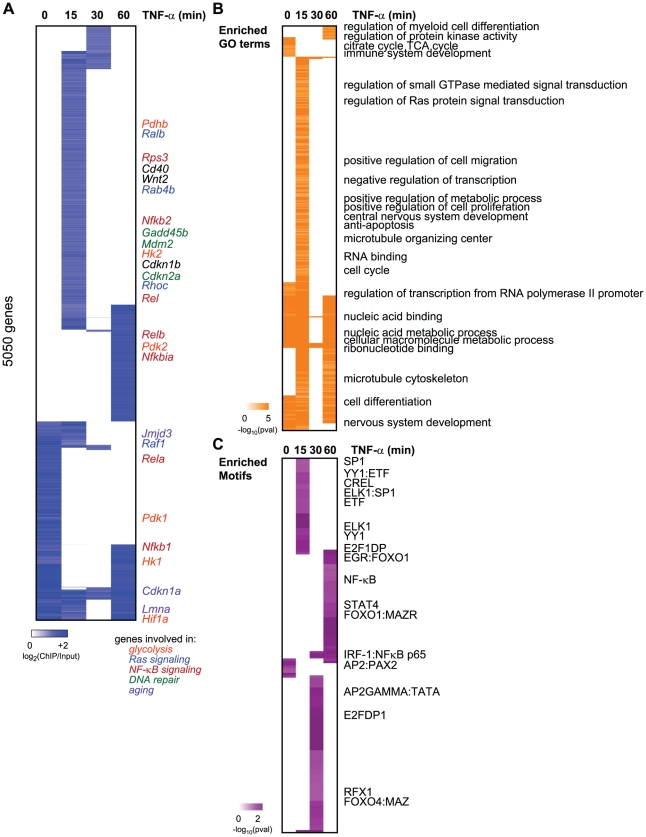
Dynamic relocalization of Sirt6 genome-wide upon stress-signaling. (A) Endogenous Sirt6 targets the promoter of 5050 genes in the presence or absence of TNF-α. The heatmap displays the binding data as the degree of enrichment of ChIP DNA over total genomic DNA. (B) Shown are the gene ontologies (GO) terms significantly induced (pval<.05, Benjamini) among Sirt6 targets in wild-type MEFs following treatment with TNF-α. Intensity of orange is representative of the degree of significance. (C) Shown are the motif modules significantly induced (pval<.05) among Sirt6 targets in wild-type MEFs following treatment with TNF-α. Intensity of purple is representative of the degree of significance [-ln(pval)].

We hypothesized that Sirt6 is a stress-responsive chromatin modifier, and that Sirt6 itself may relocalize to distinct target genes upon stress signaling. We used genome-scale chromatin immunoprecipitation (ChIP)-chip assays with high-density oligonucleotide arrays to analyze the binding patterns of Sirt6 and RelA in mouse embryonic fibroblasts (MEF) before and after TNF-α addition. Because we and others have observed that histone acetylations and Sirt6 occupancy are clustered in promoter regions upstream of the transcriptional start site (TSS) [10], [12], [20], we used whole genome promoter arrays tiling 3.25 kb upstream to 0.75 kb downstream of the TSS. Wild-type MEFs were treated with TNF-α for 0, 15, 30 or 60 minutes, and chromatin was immunoprecipitated using an antibody recognizing Sirt6. *Sirt6−/−* MEFs were also similarly treated as a negative control (Figure S1A, Table S1). We identified sequences bound by SIRT6 with 90% confidence using Nimblegen's peak calling software and subtracted nonspecific targets identified in the *Sirt6−/−* MEFs. Altogether, Sirt6 can dynamically bind up to 5050 gene promoters (Figure 1A, Table S2). Sirt6 bound 1899 genes in unstimulated cells. Notably upon TNF-α signaling, Sirt6 moved away from a large percentage of these site (684 of 1899), and relocalized to a much expanded set of genes (4366). Sirt6 occupancy also showed a striking periodic pattern: Sirt6 inducibly bound to thousands of genes at 15 minutes after TNF-α treatment, disengaged most of these sites at 30 minutes, and then re-engaged but also bound new sites at 60 minutes after TNF-α treatment. Thus, Sirt6 occupancy on chromatin is surprisingly dynamic and is globally reconfigured upon a specific stimulus.

The dynamic relocalization of Sirt6 at each time point is biologically coherent and enriched for specific functions and sequence motifs. Promoters bound by SIRT6 are enriched for genes with roles in the cell cycle, regulation of small GTP-ase activity, immune system development, nucleic acid binding and anti-apoptosis (FDR<0.05, Benjamini-Hochberg test) (Figure 1B). It has been previously shown that SIRT6 negatively regulates expression of glucose homeostasis by binding promoters of glycolytic genes and deacetylating H3K9Ac [12]. Consistent with this, we observed the citrate acid TCA cycle as a function highly enriched for among the genes bound by Sirt6 at baseline. We also observe Sirt6 binding the promoters of *Pfk1* and *Ldha*, as previously reported [12]. Sirt6 bound the promoter of many other glycolysis-related genes including *Pdk1*, *Pdk2* and *Hif1α*. In addition, Sirt6 has been shown to be required for Ras-mediated epigenetic silencing of the pro-apoptotic *Fas* gene [21]. Interestingly, we find that our TNF-α-induced Sirt6 targets are enriched for genes involved in regulation of Ras protein signal transduction. These genes include *Ralb*, *Raf1*, *Rhoc*, *Rab4b* and *Rab30*, *Rasgrp2* and *Nkiras1*.

We next searched for transcription factor binding motifs enriched for among SIRT6 bound promoters using motif module map [19] (Figure 1C). The NF-κB RelA motif along with other three other NF-κB motifs were significantly enriched. Consistent with our previous genes expression analyses revealing *Nfkbia, Nfkb1,* and *Nfkb2* as candidate target genes coregulated by RelA and Sirt6, these genes showed inducible SIRT6 binding to these targets upon stimulation with TNF-α [10]. Interestingly, we also detect the SP1, STAT1/3, ELK1, E2F1 and FOXO1/4 as highly enriched motifs (p<0.05, hypergeometric distribution).

### RelA occupancy genome-wide shows similar dynamics as Sirt6

**Figure 2:**
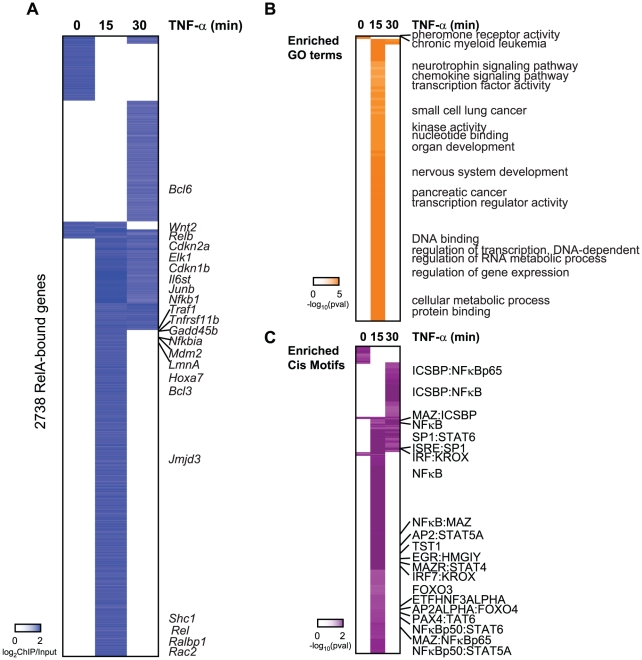
Binding of RelA resembles the occupany pattern of Sirt6. (A) Endogenous RelA targets the promoters of 2738 genes in the absence or presence of TNF-α. The heatmap displays the binding data as the degree of enrichment of ChIP DNA over total genomic DNA. (B) Shown are the gene ontologies (GO) terms significantly induced (pval<.05, Benjamini) among RelA targets in wild-type MEFs following treatment with TNF-α. Intensity of orange is representative of the degree of significance. (C) Shown are the motif modules significantly induced (pval<.05) among RelA targets in wild-type MEFs following treatment with TNF-α. Intensity of purple is representative of the degree of significance [-ln(pval)].

To compare the Sirt6 occupancy profile with that of NF-κB, we mapped RelA occupancy by ChIP-chip after TNF-α treatment for 0, 15, or 30 minutes. NF-κB is normally found in the cytoplasm, but upon TNF-α stimulation, NF-κBis transported to the nucleus where it binds to the promoter of target genes. Activation of NF-κBexhibits oscillatory behavior when stimulated by TNF-α [22]–[23]. Further studies have revealed that within a given population of cells, not all cells respond to TNF-α and at high doses of TNF-α (>0.5 ng/mL), NF-κBprotein peaks in the nucleus 20 minutes post stimulation [24]. We found that the 60 minute time point for RelA showed variable ChIP signal, possibly due to loss of synchrony RelA oscillation [10], [24], and for these reasons we did not analyze this time point by ChIP-chip. *RelA−/−* MEFs were also similarly treated as a negative control (Figure S1B). Altogether, RelA was found to occupy a total of 2738 genes with 80% confidence (Figure 2A). RelA bound 13% of genes in unstimulated cells and 91% of the 2738 genes in the presence of TNF-α. With our method, we were able to confirm the binding of RelA to 110 genes previously identified to be bound by RelA [25]–[26]. These genes include *Bcl3*, *Bcl6*, *Rac2*, *Traf1*, *Il6st*, *Hoxa7*, *Elk1*, *Junb* and *Relb*. It should be noted that previous studies interrogating genome-wide binding of RelA to DNA were carried out in a different species and in response to LPS, in which the periodic NF-κB response may be partially obscured by antiphasic feedback loops [22]–[23],. Importantly, RelA occupancy also peaks at 15 minutes and redistributes at 30 minutes after TNF-α treatment in a very similar pattern to Sirt6 occupancy.

RelA target genes are enriched for genes with similar functions and motifs as SIRT6 targets. These include chemokine signaling, immune system development, nervous system development, and pathways in cancer (p<.05, Benjamini) (Figure 2B). Specific cancer pathways that were highly enriched for by our RelA targets include chronic myeloid leukemia, glioma, small cell lung cancer, pancreatic cancer. As anticipated, the NF-κB RelA motif along with 14 instances of gene sets containing related NF-κB motifs were significantly enriched (Figure 2C), highlighting the high quality of our ChIP-chip technique. We also detect motifs containing ICSBP, MAZ, SP1, KROX, PAX4, STAT4/5A/6, TST1 and EGR highly enriched for (p<.05, hypergeometric mean).

### RelA drives dynamic relocalization of Sirt6 genome-wide

The direct physical interaction of RelA and Sirt6 raises the hypothesis that RelA can drive the stress-responsive relocalization of Sirt6 genome-wide, thereby allowing Sirt6 to deacetylate histones occupying the promoters of NF-κB target genes. The importance of RelA in Sirt6 function has been demonstrated by the ability of *RelA* haplo-insufficiency to rescue the lethality and degenerative phenotypes of *Sirt6* knockout mice [10]. While genes occupied by Sirt6 independently of RelA may well be interesting from other perspectives [also suggested by our data (Figure 1C)], we reasoned that genes under the joint regulation of Sirt6 and RelA must be central to Sirt6 function in promoting organismal longevity and health. Joint occupancy by Sirt6 and RelA should be an efficient approach to prioritize functional target genes among the numerous Sirt6-bound genes.

**Figure 3:**
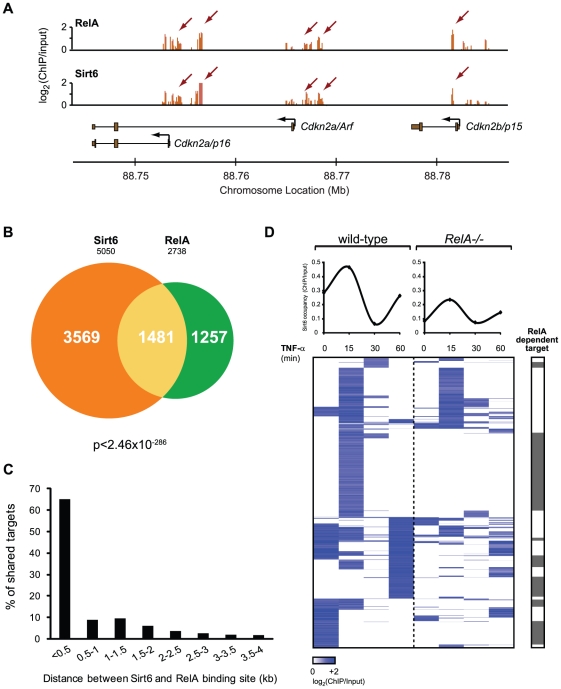
RelA drives dynamic relocalization of Sirt6. (A) Example of ChIP-chip data for Sirt6 and RelA show substantial similarity. (B) Venn diagram representing the overlap of Sirt6 and RelA promoter bound regions. (C) RelA and Sirt6 bind shared targets within 500 bp of each other. The distance between RelA and Sirt6 binding sites and the percentage of shared targets with the corresponding distance is plotted. (D) Sirt6 occupancy at promoters shared by RelA is largely dependent on RelA. Shown is a heat map displaying the ChIP binding data in wild-type and *RelA−/−* MEFs following TNF-α treatment for the indicated times, for 1481 gene promoters shared by RelA and Sirt6. The average binding of Sirt6 for each sample (column) is plotted above. The grey bar indicates that Sirt6 binding is completely abrogated in *RelA−/−* samples. 49% of shared Sirt6 targets are dependent on RelA.

Indeed, RelA and Sirt6 significantly colocalized to the same genomic sites (Figure 3A, 3B). RelA and Sirt6 bound to 2738 and 5050 promoters, respectively, of which 1481 promoters were in common (p<2.5×10^−286^, hypergeometric distribution) (Figure 3B, Table S3). This striking number represents 54% and 29% overlap of all RelA and Sirt6 targets, respectively, and suggests that Sirt6 and RelA collaborate to regulate a large fraction of their target genes. Moreover, when Sirt6 and RelA co-occupied the same promoters, Sirt6 and RelA bound at sites less than 500 base pairs apart—the shearing size of our chromatin fragments and limit of resolution— for the majority of targets (65%, Figure 3C). To provide direct genetic proof of the requirement of RelA for Sirt6 mobilization, we performed Sirt6 ChIP-chip in *RelA−/−* MEFs following induction with TNF-α for 0, 15, 30 and 60 minutes. Sirt6 and RelA do not influence each other's protein level [10]. We compared the binding of Sirt6 to the shared Sirt6 and RelA targets in *RelA−/−* cells to wild-type cells. Consistent with our hypothesis, Sirt6 occupancy was completely abrogated for 49% of shared targets (Figure 3D), most of which are inducibly targeted to chromatin by TNF-α treatment in wild-type cells. These genes include *Nfkbia*, *Gadd45b*, *RelB*, *Ralbp1*, *Cdkn2a* and *Cdkn1b*. For a number of canonical NF-κB target genes including *Nfkb1*, *HoxA7* and *Rel*, however, Sirt6 binding was reduced but not abrogated, suggesting that other NF-κB family members can potentially compensate and target Sirt6 to the promoters of NF-κB targets. In genes bound by Sirt6 but not RelA, Sirt6 occupancy at many genes is also altered in *RelA−/−* cells, possibly due to indirect effects of RelA (Figure S2). Together, these data suggest that a substantial part of dynamic Sirt6 localization to chromatin is driven by RelA.

### Gene expression consequences of interplay between Sirt6 and RelA

**Figure 4:**
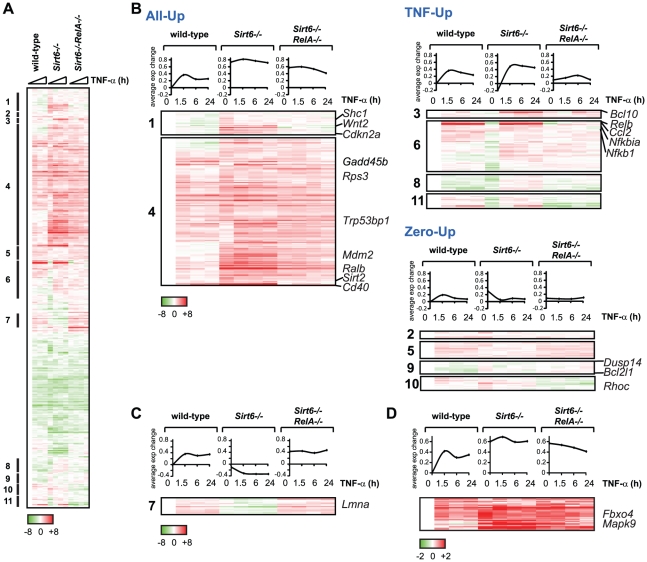
Gene expression consequences of interplay between RelA and Sirt6. (A) Targeting of promoters by RelA and Sirt6 impacts gene expression. Shown is the gene expression analysis of 480 targeted by both RelA and Sirt6 following TNF-α treatment for the indicated times. (B) 301 genes exhibited an expression pattern consistent with antagonistic regulation by RelA and Sirt6. Based on the differential expression observed in *Sirt6−/−* cells, we separated these genes into 3 distinct classes called “All Up,” “TNF-Up or “Zero Up,”. (C) A surprising class of genes, in which expression is reduced upon *Sirt6* knockout, behaves contrary to our model of Sirt6 repression of NF-κB target genes. *Lmna*, a gene thought to prevent aging, is in this class. (D) Class of genes in which Sirt6 targeting to promoters is inhibited by RelA.

We next measured the global gene expression patterns of wild-type, *Sirt6−/−* or *Sirt6-/-RelA−/−* cells in the temporal response to TNF-α. We focused on genes with promoters bound by Sirt6 and RelA in order to understand the consequences of Sirt6 and RelA binding. A further goal is to identify candidate genes that may drive the aging process because genetic evidence suggests a critical role for genes jointly regulated by Sirt6 and RelA [10]. We were able to detect expression in 480 out of the 1481 targets shared by RelA and Sirt6 (Figure 4A). Out of these 480 genes, 301 genes exhibited an expression pattern consistent with antagonistic regulation by RelA versus Sirt6. Specifically, expression of these genes was elevated in *Sirt6−/−* cells compared to wild-type and correspondingly reduced in *Sirt6−/− RelA−/−* cells. Hierarchical clustering of the gene expression patterns organized the genes into three distinct classes based on their differential expression in *Sirt6*−/− cells (Figure 4B). The first class, termed “All-Up”, showed elevated gene expression in *Sirt6−/−* cells at baseline without TNF-α treatment and at every single time point after TNF-α-treatment. All-Up includes genes such as candidate aging regulators *Shc1*(encoding p66) [27], *Cdkn2a* (encoding the cell cycle inhibitor p16 that increases in expression with age) [28]–[29], *Wnt2* [30]–[31], and stress responsive genes *Gadd45b* [32] and *Mdm2* [33]. Adler et al. previously demonstrated that age-dependent accumulation of p16 protein in vivo requires ongoing NF-κB activity [19]. Our data further reinforce this observation, and suggest that p16 protein accumulation is due in part to direct RelA-mediated transcription of *Cdkn2a* (Figure 3A, Figure 4B).

In the second class termed “TNF-Up”, expression in *Sirt6*−/− cells is increased only upon stimulation with TNF-α, demonstrating the importance of stress signaling to elucidate transcriptional alterations in *Sirt6−/−* cells. The TNF-Up class includes genes such as *Bcl10*, *Relb*, *Ccl2* and *Nfkbia* that encode both positive and negative feedback mechanisms to NF-κB signaling [17]. Third, the “Zero-Up” class is a smaller set of genes that showed increased gene expression in *Sirt6−/−* cells only in the absence of TNF-α, and this aberrant pattern is reversed in *Sirt6−/−Rela−/−* cells. This class includes genes encoding several signaling proteins such as protein phosphatase *Dusp14*, anti-apoptotic gene *Bcl2l1* and actin regulator *Rhoc.* Within both All-Up and Zero-Up classes, there is a small subset of genes that are normally repressed upon treatment with TNF-α. In *Sirt6−/−* cells, however, stimulation with TNF-α induces expression, suggesting that Sirt6 is normally responsible for preventing expression of these genes in the presence of TNF-α. These genes include *Sdc2*, *Tinagl*, *Pkia* and *Tnfrsf11b*.

**Figure 5:**
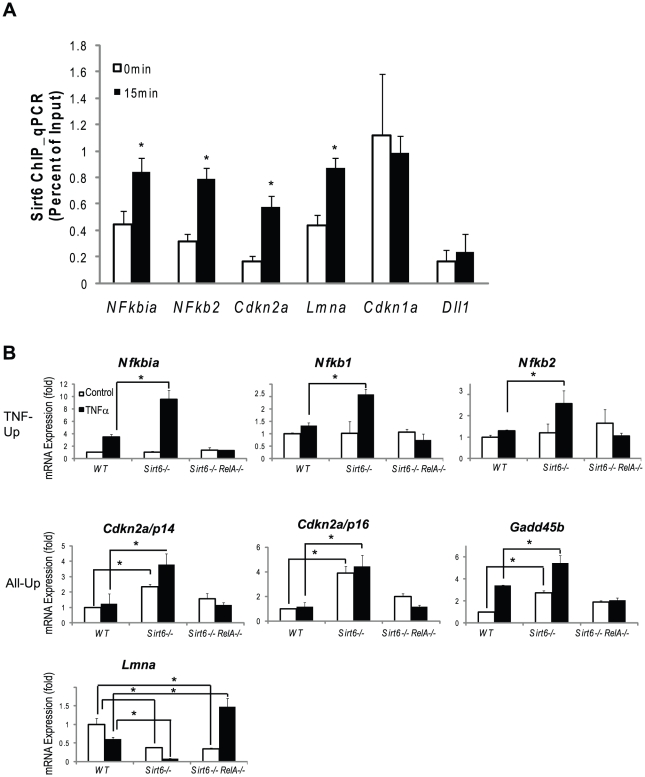
Validation of Chip-chip data and gene expression data. (A) SIRT6 is recruited to the promoters of canonical NF-κB target genes (*Nfkbia*, *Nfkb1*, *Nfkb2*) and to the promoters of genes involved in aging (Cdkn2a/p16 and Lmna). Independently derived wildtype MEFs were treated with TNF-α (20 ng/ml) for 0 or 15 minutes. ChIP with α-Sirt6 antibodies was preformed and percent Sirt6 occupancy is shown relative to input as measured by qPCR. Mean ±S.E. of three technical replicates is shown. (B) Gene expression validation by qRT-PCR. TNF-Up genes demonstrate increased expression in Sirt6 −/− cells in response to TNF-α which is abolished in Sirt6 RelA −/− cells. All-Up genes demonstrate increased expression in Sirt6 −/− cells in the presence and absence of TNF-α which is attenuated inSirt6 RelA −/− cells. Lmna expression is repressed in Sirt6 −/− cells and derepressed in Sirt6 RelA −/−in response to TNF-ã Wildtype, Sirt6 −/− and Sirt6 RelA−/− MEFs were treated with TNF-a (20 ng/mL) for 0 and 1.5 hrs. Quantitative Taqman real time RT-PCR of the indicated mRNAs is shown normalized to GAPDH levels. Mean ± S.D. is shown. *p<0.05, student's *t*-Test.

Furthermore, we found two interesting classes of genes which behave in a manner contrary to our model of Sirt6 repression of NF-κB target genes (Figure 4C, 4D). In one class, gene expression is repressed in *Sirt6−/−* cells compared to wild-type cells, and repression is relieved in *Sirt6−/−RelA−/−* cells to an even greater extent than in wild-type cells (“Inverse”, Figure 4C). Notably, one such gene is *Lmna* (encoding lamin A), which has been previously linked to aging [34]. In the second class of genes, dynamic localization of Sirt6 impacts gene expression, as Sirt6 is recruited to and away from its binding sites. Sirt6 occupies the promoters of a diverse set of genes in the basal state and its occupancy is linked with transcriptional repression. Upon the addition of TNF-α, Sirt6 is largely redistributed to new promoters, in part through its direct interaction with RelA [10]. As a result of relocation of Sirt6 to new targets, TNF-α treatment may induce gene de-repression. We identified a set of genes that fulfill these criteria and are bound by bound by Sirt6 only in the absence of TNF-α (Figure 4D). These genes include *Mapk9* and *Fbxo4*. Since RelA inhibits Sirt6-dependent repression of these genes, they show an opposite epistatic relationship: They are derepressed in *Sirt6−/−* cells, but are not reverted in *Sirt6−/−RelA−/−* cells (Figure 4D, Figure 5). Meanwhile, TNF-α induces stabilization of Sirt6 at the promoters of genes including *p16*, *Gadd45b* and *Nfkb2*, titrating them away from the promoters of targets that are de-repressed.

Next, we validated our ChIP-chip results on a subset of known and novel Sirt6 target genes. Independently derived wildtype MEFs were stimulated with TNF-α for 0 and 15 minutes and Sirt6 ChIP was performed using a microfluidic device [35] followed by quantitative PCR to interrogate Sirt6 occupancy of the indicated promoters (Figure 5A). In response to TNF-α stimulation, Sirt6 was inducibly recruited to the promoters of canonical NF-κB target genes *Nfkbia* and *Nfkb2*. Sirt6 was also recruited to the promoters of two novel loci, *Cdkn2a* and *Lmna*, in response to TNF-α stimulation (p<0.05). Additionally, we found that Sirt6 is located at the promoter of *Cdkn1a* independent of TNF-α stimulation. As a negative control, we did not detect Sirt6 at the promoter of *Dll1*, consistent without ChIP-chip results. We also demonstrate the reproducibility of these experiments by perfoming ChIP on two additional independently derived non-littermate wildtype MEF lines on two targets, *Nfkbia* and *Nfkb2* and show the inducibility of Sirt6 recruitment to the promoters of these genes in response to TNF-α stimulation (Figure S1).

We validated our expression array data by examining mRNA expression in wild type, *Sirt6−/−* and *Sirt6−/−* *RelA−/−* double knockout MEFs quantitative reverse transcription PCR. In wildtype cells, TNF-α stimulation led to the inducible expression of the “TNF-Up” genes, *Nfkbia, Nfkb1* and *Nfkb2* (p<0.05 vs uninduced for each, student's *t*-test). In Sirt6 knockout cells, no difference was seen at baseline, but TNF-α stimulation led to ∼2-fold greater induction of these genes when compared to wildtype at time 1.5 hours (p<0.05). In *Sirt6 RelA* double knockout cells the inducibility of the “TNF-Up” genes was abolished, demonstrating that these gene expression changes are dependent on RelA (Figure 5B).

Next, we tested genes in the “All-Up” class, including candidate aging regulator, *Cdkn2a/p16*, the coregulated transcript, *Cdkn2a/p14*, and stress response gene, *Gadd45*. Here we find that *Cdkn2a/p16* and *Cdkn2a/p14* are very weakly induced in response to TNF-α in wildtype cells. However, both of these transcripts are induced in the *Sirt6* knockout cells in the absence of TNF-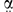 and Cdkn2a/p16 can be superinduced by TNF-α in Sirt6−/− cells (p<0.05 for each comparison). Again, the inducibility of both transcripts was abolished in *Sirt6−/−RelA−/−* double knockout cells. On the other hand, *Gadd45b* expression is upregulated three-fold both in response to TNF-α in wildtype cells and in *Sirt6* knockout cells at time 0 (p<0.05). Furthermore, induction with TNF-α in *Sirt6* knockout cells led to a further two-fold superinduction of *Gadd45b* gene expression when compared to *Sirt6* knockout at time 0 hours (p<0.05). In double knockout cells, *Gadd45b* is no longer responsive to TNF stimulation, indicating that the induction requires NF-κB (Figure 5B).

Finally, we tested the progeria causing gene, *Lmna*, which falls into the “inverse” category of genes that are repressed in *Sirt6* knockout cells. In wildtype cells, TNF-α stimulation led to a 40% reduction in *Lmna* expression. In *Sirt6* knockout cells at time 0, *Lmna* expression was reduced by 60% and was reduced about 10-fold after TNF-α stimulation when compared to wildtype at time zero (p<0.05). In *Sirt6*−*/*− *RelA−/−* cells at time 0 gene expression was reduced to by 60% as in *Sirt6* knockout cells, but was induced nearly 1.5 fold with TNF-α stimulation (p<0.05) (Figure 5B). Mutations in *Lmna* cause Hutchinson-Gilford progeria syndrome (HGPS) [36], a disorder with clinical features of premature aging. Normal aging is associated with accumulation of a spliced isoform of *Lmna* that impedes nuclear function. *Lmna* expression is decreased in *Sirt6−/−* cells but induced in *Sirt6−/−RelA−/−* cells in response to TNF-α, consistent with a role for Sirt6 in modulating NF-κB-dependent expression to prevent aging. The *Lmna^L530P/L530P^* mice display a progeroid phenotype that strikingly resembles the phenotype of *Sirt6−/−* mice: reduced size, loss of subcutaneous fat, reduced bone mineral density and lethality by 4–5 weeks of age. These results raise the possibility that some of the characteristics of *Sirt6−/−* mice could be driven by deregulated *LmnA* expression. Since *Lmna* expression is reduced rather than enhanced in Sirt6 knockout, however, Sirt6 and RelA may regulate a factor that represses *Lmna* expression. Whether Sirt6 alters Lmna splicing, lamin A processing, or nuclear architecture remains to be determined.

**Figure 6:**
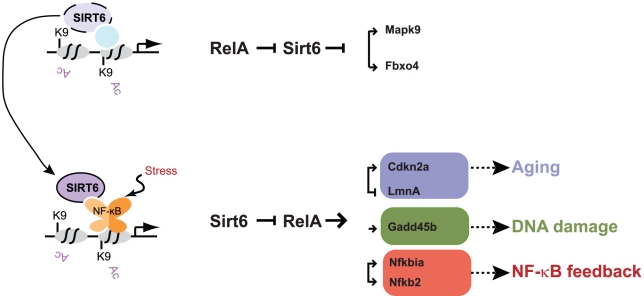
Model for Sirt6 regulation of targeting and gene expression. Sirt6 occupies the promoters of a wide array of genes, likely in conjunction with other transcription factors. We propose that upon the addition of a stress such as TNF-α, Sirt6 redistributes to promoters via its interaction with RelA. This redistribution enables derepression of a subset of genes (e.g. *Mapk2*, *Fbxo4*) and induction of repression of genes involved in pathways such as aging (*Cdkn2a*, *Lmna*), DNA damage response (*Gadd45b*), and NF-κB feedback (*Nfkbia*, *Nfkb2*). The experience of NF-κB activation may influence subsequent rounds of stress response by creating a new basal state of Sirt6 occupancy.

Together these data demonstrate that the interplay of chromatin localization of Sirt6 and RelA regulates the expression of many genes, many of which have roles in regulating aging, stress response and NF-κB feedback, via both direct and indirect mechanisms (Figure 6).

## Discussion

Deletion of Sirt6 results in a premature aging-like degenerative syndrome in mice, and heterozygosity of the NF-κB subunit RelA can rescue the degenerative and early lethality phenotype [10]–[11]. We and others also independently identified NF-κB as a positive regulator of aging-related gene expression programs and phenotypes [19], [37]–[38]. These findings suggest a model in which aging is caused in part by elevated NF-κB-dependent gene expression. In contrast, Sirt6, via its H3K9Ac deacetylase activity, restrains NF-κB dependent transcription and promotes longevity. Because this model is based on chromatin analysis of a small number of genes, its generality and the relevant target genes were not clear. Here, we have coupled genome-scale profiling of RelA- and Sirt6-bound sites with global gene expression analysis to identify direct targets of RelA and Sirt6. Sirt6 occupies the promoters of 54% of RelA targets. Of these genes, we demonstrate Sirt6-dependent repression of basal and/or TNF-α-induced expression of hundreds, many of which have been shown to regulating aging and longevity (Figure 6).

### Dynamic localization of Sirt6

Dynamic relocalization of sirtuins is an important feature of both yeast Sir2 and mammalian Sirt1. Yeast Sir2 represses recombination or transcription at telomeres, silent mating type loci and ribosomal DNA. Upon the addition of DNA damage agents, Sir2 leaves telomeric sites and relocates to sites of DNA damage [39]. Similarly, Sirt1 was recently shown to occupy a diverse set of genes in the basal state, but in response to DNA damage, Sirt1 redistributes to these sites of DNA damage [40]. These findings are also consistent with recent work documenting inducible recruitment of Sirt6 to sites of DNA damage [8].

We now show that Sirt6 binds thousands of promoters in the mouse genome and that similar to RelA, this binding pattern is largely reconfigured in response to stress induced by TNF-α. 29% of Sirt6 targets are shared by RelA, and of these shared targets, 49% require RelA for Sirt6 occupancy. This suggests that in response to TNF-α or one of the many stressors that activate NF-κB, the stress-responsive nature of Sirt6 binding could be driven by its interaction with the stress-responsive transcription factor RelA. However, Sirt6 targets a large number of genes in the absence of RelA, which begs the question of whether another NF-κB family member or related transcription factor may also contribute to Sirt6 localization (Figure S2). In addition, the NF-κB pathway is involved in crosstalk with several pathways, including JNK, STAT3 and Foxo3a, and, these pathways will be altered in the absence of RelA [41]. It will be interesting to determine whether other stress-responsive transcription factors exist to guide the relocalization of Sirt6 and other Sir2-related deacetylases upon distinct stress signals such as DNA damage. Our identification of specific transcription factor motifs at Sirt6 bound sites provide a logical starting point for other regulators of Sirt6 localization.

### Impacts on gene expression

Sirt6 and RelA share a large number of direct targets and co-regulate expression of these genes. Genes under the joint control of Sirt6 and RelA include several key regulators of aging, including *CDKN2A* (encoding p16), *Shc1*, Wnt, and *LMNA*. The interplay of Sirt6 and RelA occupancy can shape several patterns of stimulus-dependent gene expression programs, as revealed in single and double knockout MEFs. Although MEF cultures can be heterogeneous, we have previously shown that the NF-κB driven gene expression program is independent of anatomical origin and is instead responsive to stress and aging [19]. Further studies revealed that Sirt6 and RelA regulate a similar set of genes by acute RNAi depleton through a similar relationship [10].

While the majority of genes with joint occupancy of Sirt6 and RelA showed the expected epistatic relationships (Sirt6 --| RelA ◊ gene), there is a surprising diversity of stimulus-dependent NF-κB responses when Sirt6 is absent (Figure 4). In other words, chromatin regulation by Sirt6 is important to maintain precise coordination among NF-κB target genes. Notably, loss of coordinate expression of NF-κB target genes is a defining characteristic of aging tissues [37]. Understanding the context of additional transcription factors and chromatin states at these genes may help to explain these quantitative differences. Furthermore, we provide evidence that the stress-dependent movement of Sirt6 away from its basal binding sites can also de-repress gene expression, yielding a reversed regulatory hierarchy (RelA --| Sirt6 --| gene, Figure 6). This latter class of genes was only appreciated due to the comprehensive epigenomic studies and integrative analyses with global gene expression patterns. One important caveat of our study is that all of our measurements are based on populations of cells, and it is now clear that variation in behavior on a cell-by-cell basis (i.e. noise) also modulates the overall NF-κB response [23]-[24]. While the observed changes in promoter occupancy and gene expression strongly indicate that target genes are directly regulated by RelA and Sirt6, some of the observed changes may also be due to indirect effect of Sirt6 or RelA inactivation. Future studies addressing genome-wide binding of NF-κB family members as well as dynamic gene expression with high temporal precision and in single cells shed light on these outstanding questions.

## Materials and Methods

### Ethics statement

All animals were handled in strict accordance with good animal practice as defined by the relevant national and/or local animal welfare bodies. All animal work was approved by the Stanford University Institutional Animal Care and Use Committee.

### Antibodies and cell lines

Antibodies specific for RELA (Abcam), SIRT6 (Abcam) and Actin (Santa Cruz) are from the indicated sources. MEFs were generated from 13.5-day-old embryos using standard methods and propagated in DMEM (Invitrogen) plus 15% FBS. MEFs were passaged a total of four times before TNF-α treatment.

### Chromatin immunoprecipitation (ChIP)

Table S1 provides a summary of all data generated and analyzed in this paper. *RelA −/−* and *Sirt6 −/−* MEFS were generated from littermate embryos derived on the same day as wildtype embryos. Biological replicates from wildtype embryos were generated from independently derived non littermate embryos, grown at separate times and treated with TNF-α on separate days. See Table S1 for details on number of independent experiments. Cells were treated with TNF-α (20 ng/ml) for the indicated times. DNA was cross-linked for 10 minutes with 1% formaldehyde and stopped in 0.125 M glycine. Purified chromatin was sonicated to ∼500 bp using the Bioruptor (Diagenode, Inc) and incubated with the indicated antibodies as previously described [10]. Following reverse cross-linking and RNase treatment, DNA was purified with the Ziagen Mini-elute Reaction Cleanup Kit and amplified using the Whole Genome Amplification kit (Sigma) as described by the manufacturer.

### ChIP-chip assays

Each amplified DNA sample was labeled according to the manufacturer's ChIP-chip protocol (Nimblegen). Briefly, each DNA sample (1 µg) was denatured in the presence of Cy5- or Cy3-labeled randon nonamers and incubated with 100 units (exo-) Klenow fragment (NEB) and dNTP mix (6 mM each in TE buffer) for 2 hours at 37°C. Reactions were terminated by addition of 0.5 M EDTA (pH 8), precipitated with isopropanol, and resuspended in water. 12 µg of Cy5-labeled ChIP sample and 6 µg of Cy-3 labeled total sample were mixed, dried and resuspended in 40 µL of buffer including hybridization buffer (Nimblegen), alignment oligonucleotides (Nimblegen) and Component A (Nimblegen). The labeled DNA samples were next denatured and hybridized to “MM8” arrays overnight at 42°C. Samples were co-hybridized with input DNA as a reference, and the microarrays (Nimblegen) contained probes tiling a total of 4 kilobases of 21,249 mouse promoters. Fluorescence intensity raw data were obtained from scanned images of the oligonucleotide tiling arrays using NIMBLESCAN 3.0 extraction software (Nimblegen). For each spot on the array, log2-ratios of the Cy5-labeled test sample versus the Cy3-labeled reference sample were calculated. To normalize across samples, the biweight mean of this log2 ratio was subtracted from each point. ChIP-chip time courses were done twice or more for most time points; representative data from single time courses are shown.

### ChIP-chip data analysis

Identification of targets: Raw Chip-chip data were normalized using RMA normalization algorithm in NimbleScan software. Peaks were called out using NimbleScan peak-calling package. We determined whether canonical NF-κBtargets identified by ChIP-seq [12], [42] and previously identified Sirt6 targets [10] were detected by for different threshold values (Figure S3). For RelA, an FDR ≤0.1 failed to retrieve 414 of 980 known canonical targets while an FDR ≤0.2 retrieved all of them. For Sirt6 an FDR ≤0.1 retrieved all the known Sirt6 targets previously identified [10]. An FDR ≤0.2 increased the number of Sirt6 targets by 5547 additional genes, of which 627 (11.3%) are also bound by RelA with an FDR ≤0.2. Compared to the 29% RelA co-occupancy with a Sirt6 FDR ≤0.1, this analysis shows that the higher confidence Sirt6 target genes tend to be co-occupied with RelA. Therefore, for Sirt6, data for all peaks with an FDR ≤0.1 were included and for RelA, data for all peaks with an FDR ≤0.2 were included. Promoter regions were defined as +/−4000 bp of RefSeq genes (mm8). Sirt6 and RelA Chip target genes were obtained by overlapping significant peaks with promoter coordinates in Galaxy, using functions of “getflanks”, “intersect”, “substract” and “join”. To determine the binding sites for Sirt6 and RelA, the list of targets was filtered to include only genes that were targets in wild-type cells and not in the negative control knockout cells (*Sirt6−/−* or *RelA−/−*, respectively).

Motif module map: To analyze cis-regulatory motifs enriched among Sirt6 and RelA direct targets, we identified promoters of genes bound by Sirt6 or RelA and tested for their enrichment (*p*<0.05, hypergeometric distribution) of sets of genes sharing the presence of cis-motifs, termed motif modules [19]. We then selected for motif modules that were induced among wild-type and *Sirt6−/−* MEFs. All motifs attributed to any of the five NF-κB family members are referred to as “NF-κB”.

GO term and KEGG pathway analyses: Functional annotations were performed using the program Database for Annotation, Visualization, and Integrated Discovery (DAVID). We used a Benjamini threshold of <0.05.

### ChIP-qPCR

Sirt6 antibody was conjugated to magnetic Protein A beads (Invitrogen) using the manufacturer supplied protocol. Prior to IP, a volume of sonicated chromatin equivalent to four times the amount used for the IP was reserved as the input sample. Three technical replicates were done for each time point shown, using an equivalent of 10,000 cells of chromatin each time. ChIP was performed using sonicated chromatin on an automated microfluidic device (modified from that previously described [35], to be detailed in a future publication), for a total of two hours on a 4°C thermocycler block, with constant mixing. After IP, RIPA buffer (10 mM Tris-HCl pH 7.5, 1 mM EDTA, 0.5 mM EGTA, 1% Triton X-100, 0.1% SDS, 0.1% Na-deoxycholate, 140 mM NaCl) was used to wash the beads for 10 minutes, following which the beads were then eluted into thin-walled PCR tubes with a minimal amount of Dulbecco's phosphate buffered saline (DPBS). These PCR tubes were then immediately placed on a magnet and the DPBS removed with a pipette leaving only the magnetic beads. The DNA was then purified from the beads using the Chelex (Bio-Rad) resin extraction method described previously [43]. An ethanol precipitation was done on the input sample by adding 250 ul of 100% ethanol (Sigma-Aldrich), 2 ul of carrier glycoblue (Invitrogen), and 16 ul of 5 M NaCl to the sample and precipitating at −80°C for one hour. The precipitated sample was then centrifuged at 20,000 g for 15 minutes, and the supernatant discarded. The pellet was washed in 500 ul of freshly prepared and chilled 70% ethanol, and then centrifuged again at 20,000 g for 10 minutes. Finally, the supernatant was discarded and the pellet left to air dry. Once the pellet was dry, the same Chelex resin extraction was applied in parallel with the IP samples. The purified DNA was used directly in the real-time quantitative SYBR green PCR reactions. Sequences of PCR primers used are provided in the Table S4.

### Microarray-based RNA expression assays

MEFs were derived from littermate embryos. Total RNA was extracted with TRIzol (Invitrogen) from mouse embryonic fibroblasts following TNF-α treatment (20 ng/mL) for the indicated times. RNA was labeled with Cy5 and hybridized to whole genome mouse bead arrays (Illumina). Genes that were induced with an absolute detection value of 100 in any sample during the time course were selected and normalized to the untreated wild-type sample. All genes and samples were next organized by hierarchical clustering [44].

### Real-time quantitative RT-PCR

Total RNA was extracted with TRIzol. RT-qPCR was performed using total RNA (50 ng), Taqman One Step RT-PCR master mix, and one of the following Taqman assays: GAPDH (Mm99999915g1*), NFKB1 (Mm00476361_m1*), NFKB2 (Mm00479807_m1*), NFKBia (Mm00477798_m1*), Cdkn2a/p14 (Mm01257348_m1), Cdkn2a/p16 (Mm00494449_m1*), Gadd45b (Mm00435123_m1*) and Lmna (Mm00497787_g1). All Taqman reagents were from Applied Biosystems. Reactions were in triplicate for each sample and preformed a minimum of three times. Data were normalized to GAPDH levels.

### URLs

Primary microarray data are available at Stanford Microarray Database (http://smd.stanford.edu) and GEO (http://www.ncbi.nlm.nih.gov/geo/) with accession GSE28641.

## Supporting Information

Figure S1 Western analysis of Sirt6 and RelA protein levels. (A) Immunoblot of Sirt6 in wild-type and *Sirt6−/−* MEFs. (B) Immunoblot of RelA in wild-type and *RelA−/−* MEFs. (C) Two biological replicates of the Sirt6 ChIP-qPCR demonstrating that Sirt6 is recruited to the promoters of *Nfkbia* and *Nfkb2*. MEF cells independently derived from two WT mouse embryos were treated with TNF-α (20 ng/ml) for 0 or 30 minutes. ChIP with α-Sirt6 antibodies was preformed and percent Sirt6 occupancy is shown relative to input. Mean of two technical replicates is shown.
(EPS)

Figure S2 Sirt6 occupany at promoters not shared by RelA. Heat map displaying the Sirt6 ChIP binding data in wild-type and *RelA−/−* MEFs following TNF-α treatment for 3569 gene promoters that are bound by Sirt6 but not RelA. Sirt6 occupancy at many promoters in wildtype cellsis altered in *RelA−/−* cells at baseline and upon TNF-α stimulation indicating that our assay is not sensitive enough to detect co-occupancy or that RelA may exert indirect effects on Sirt6 localization.
(EPS)

Figure S3 Comparison of FDR of ≤0.1 and ≤0.2 in RelA Chip-chip signals. RelA ChIP-chip signal time course in WT and *RelA* KO MEFs using different FDRs demonstrate that an FDR of ≤0.1 fails to retrieve known and authentic RelA target genes such as *Bcl2a1a, Ill6, Cxcl1* and *Nfkbiz* while an FDR of ≤0.2 retrieves these targets.
(EPS)

Table S1 List of Experiments Preformed. Description of cell genotype, TNFα treatment time, experiment type and analysis, number of technical and biological replicates, and figure number for all experiments preformed.
(XLSX)

Table S2 List of 5050 Sirt6 targets. Gene name and NCBI Gene ID for the Sirt6 targets identified by ChIP-chip.
(XLSX)

Table S3 List of 2734 RelA targets. Gene name and NCBI Gene ID for the RelA targets identified by ChIP-chip.
(XLSX)

Table S4 List of ChIP-qPCR primers. Primer sequences used for validation of ChIP-chip.
(XLSX)
